# Synthesis, crystal structure and Hirshfeld surface analysis of [1-(4-bromo­phen­yl)-1*H*-1,2,3-triazol-4-yl]methyl 2-(4-nitro­phen­oxy)acetate

**DOI:** 10.1107/S2056989024007436

**Published:** 2024-07-31

**Authors:** Muminjon Hakimov, Shakhnoza Khozhimatova, Ilkhomjon Ortikov, Ibragimdjan Abdugafurov, Akmaljon Tojiboev

**Affiliations:** a Namangan State University, Boburshoh str. 161, Namangan 160107, Uzbekistan; b Andijan Machine Building Institute, Bobur Shox Ave 56, Andijan 170119, Uzbekistan; c Institute of the Chemistry of Plant Substances, Uzbekistan Academy of Sciences, Mirzo Ulugbek Str. 77, Tashkent 100170, Uzbekistan; d National University of Uzbekistan, University Str., 4, Tashkent 100174, Uzbekistan; e University of Geological Sciences, Olimlar Str. 64, Tashkent 100170, Uzbekistan; University of Durham, United Kingdom

**Keywords:** crystal structure, 1,2,3-triazole, click chemistry, hydrogen bonds, Hirshfeld surface analysis

## Abstract

The title compound, C_17_H_13_BrN_4_O_5_, was synthesized by a Cu_2_Br_2_-catalysed Meldal–Sharpless reaction between 4-nitro­phen­oxy­acetic acid propargyl ether and *para*-bromo­phenyl­azide, and characterized by X-ray structure determination and ^1^H NMR spectroscopy. The mol­ecules, with a near-perpendicular orientation of the bromo­phenyl-triazole and nitro­phen­oxy­acetate fragments, are connected into a three-dimensional network by inter­molecular C—H⋯O and C—H⋯N hydrogen bonds (confirmed by Hirshfeld surface analysis), π–π and Br–π inter­actions.

## Abstract 2

The title mol­ecule has a twisted conformation and is connected with its neighbours by C—H⋯O and C—H⋯N hydrogen bonds, π–π and Br–π inter­actions.

## 1. Chemical context

1,3-Dipolar cyclo­addition, a reaction between a 1,3-dipole and a dipolarophile to generate a five-membered ring, has been known since the early 20th century, following the discovery of 1,3-dipoles; its mechanism was studied and synthetic applications were developed in the 1960s, primarily through the work of Rolf Huisgen (Bertrand *et al.*, 1994 ▸; Huisgen, 1963 ▸). Meldal and Sharpless independently developed a copper(I)-catalysed version of the Huisgen cyclo­addition reaction (Tornøe *et al.*, 2002 ▸; Rostovtsev *et al.*, 2002 ▸), which earned the name of ‘click chemistry’ for its versatility. They found that only one isomer, 1,4-disubstituted 1,2,3-triazole, was formed from the cyclo­addition of terminal alkyne and organic azides under these conditions. The mechanism of the reaction and the role of the Cu^I^ salt were fully explained. Currently, 1,2,3-triazole derivatives are researched intensively because of their pharmacological and biological activity (Borgati *et al.*, 2013 ▸; Bozorov *et al.*, 2019 ▸; Faraz *et al.*, 2017 ▸; Li *et al.*, 2015 ▸). In the course these studies, we prepared the title compound **1** by the cross-ring reaction of 4-nitro­phen­oxy­acetic acid propargyl ether with *para*-bromo­phenyl­azide and characterized it by single-crystal X-ray diffraction and NMR spectroscopy.
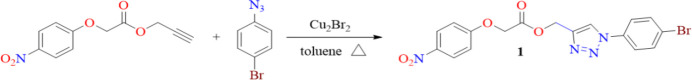


## 2. Structural commentary

**Figure 1:**
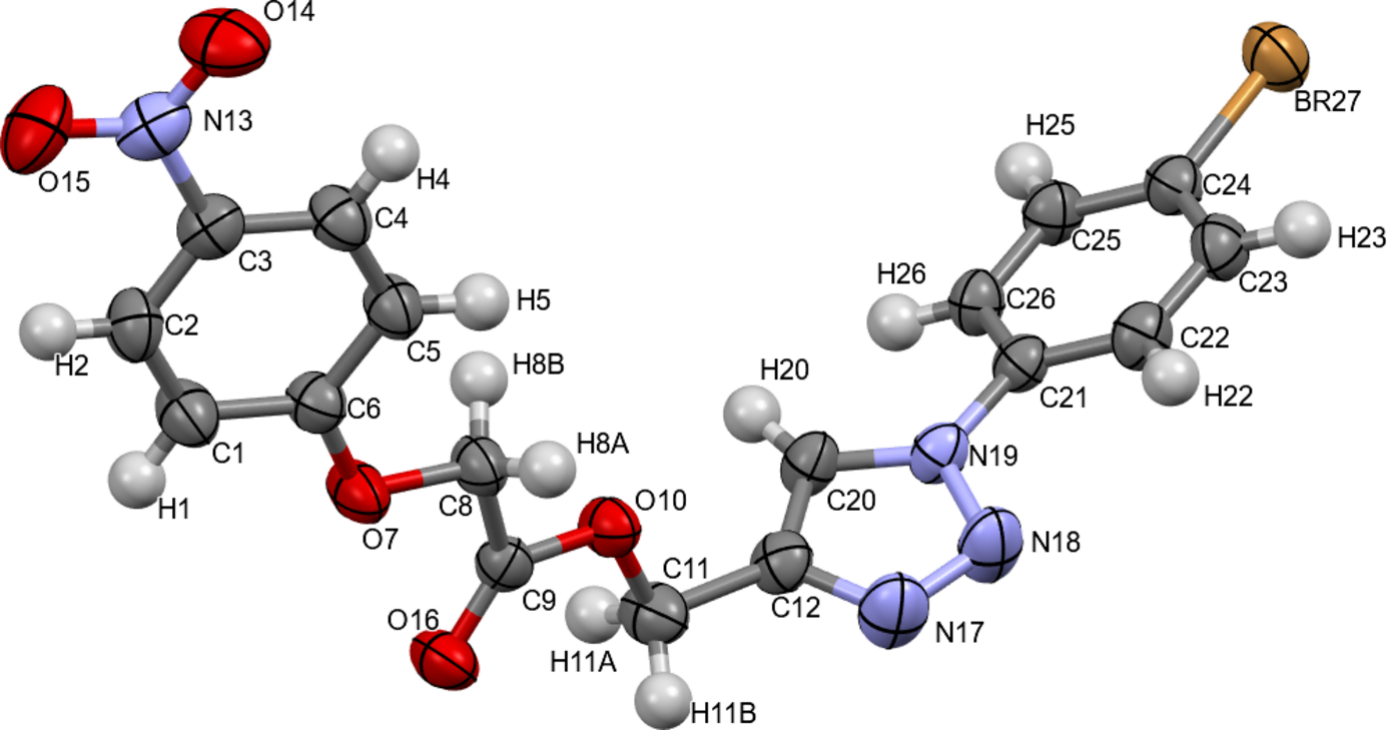
The mol­ecular structure of **1** with displacement ellipsoids drawn at the 50% probability level.

Compound **1** crystallizes in the monoclinic space group *P*2_1_/*n*, the asymmetric unit comprising one mol­ecule (Fig. 1 ▸) which contains five planar fragments, namely a bromo­phenyl group, a 1-*H*-1,2,3-triazole ring, a CH_2_OC(=O)CH_2_O bridge, phenyl and nitro groups. The inter­planar angles between adjacent fragments in this succession are 23.5 (1), 80.3 (1), 19.3 (1) and 6.0 (2)°, respectively. The N17—C12—C11—O10 torsion angle is 97.3 (3)°.

## 3. Supra­molecular features

**Figure 2:**
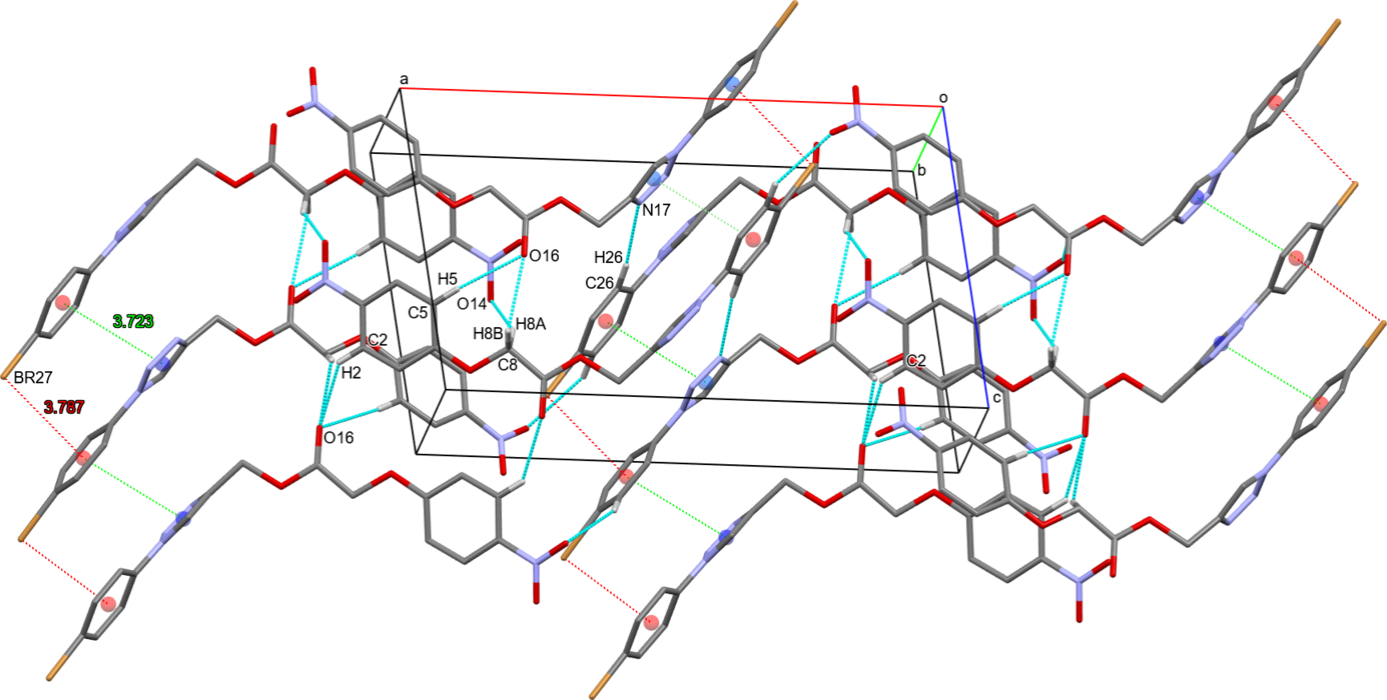
Crystal packing of **1**, showing hydrogen bonds, π-π- and Br–π inter­actions as blue, green and red dotted lines, respectively. The centroids of the triazole (*Cg*1) and brominated phenyl (*Cg*2) rings are shown by blue and red circles, respectively. H atoms not participating in hydrogen bonds are omitted.

Although the structure contains no classical strong hydrogen bonds, some inter­molecular C—H⋯O and C—H⋯N contacts (Table 1 ▸) can be identified as hydrogen bonds by Hirshfeld surface analysis (*vide infra*). They link the mol­ecules into a three-dimensional network (Fig. 2 ▸), complemented by π–π stacking between the triazole ring and the brominated phenyl ring [inter­planar angle of 8.76 (15)°, *Cg*1⋯*Cg*2 distance of 3.723 (16) Å and slippage of 0.917 Å], as well as C24—Br27⋯π inter­actions [Br27⋯*Cg*2 = 3.787 (11) Å] involving the same phenyl ring.

## 4. Hirshfeld surface analysis

**Figure 3:**
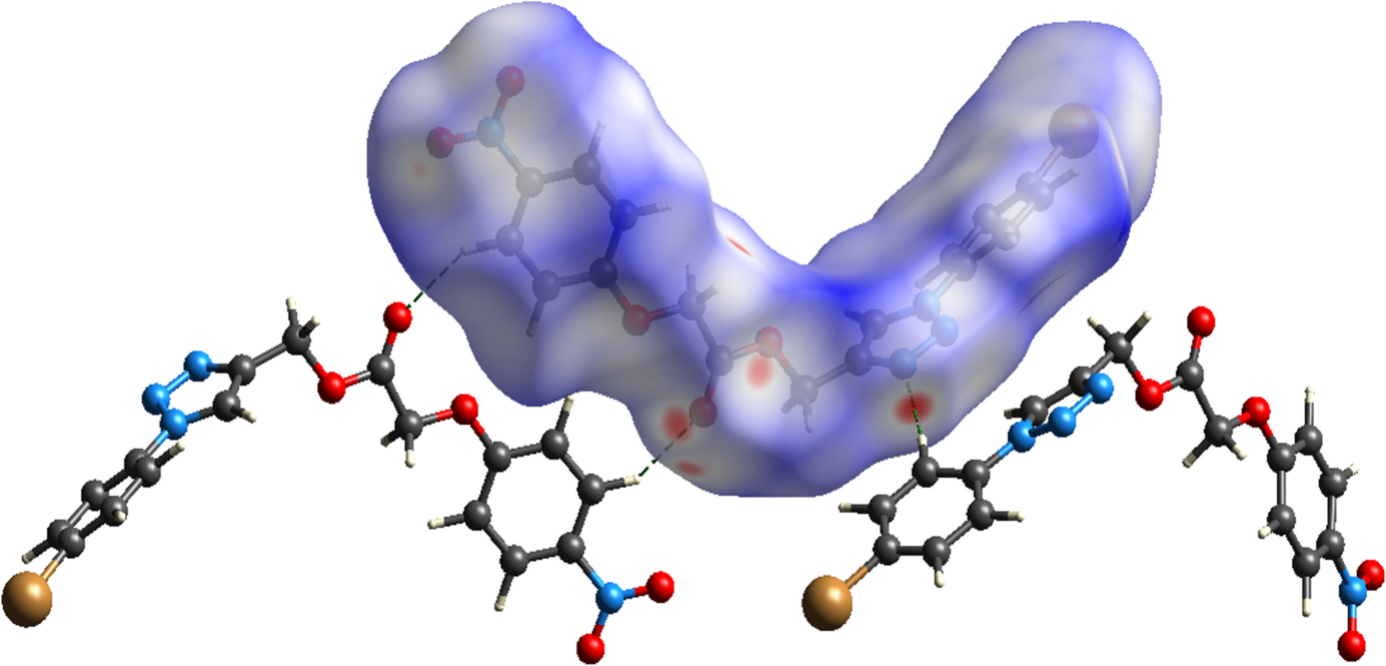
Hirshfeld surface of **1** mapped over *d*_norm_ and close inter­molecular contacts.

**Figure 4:**
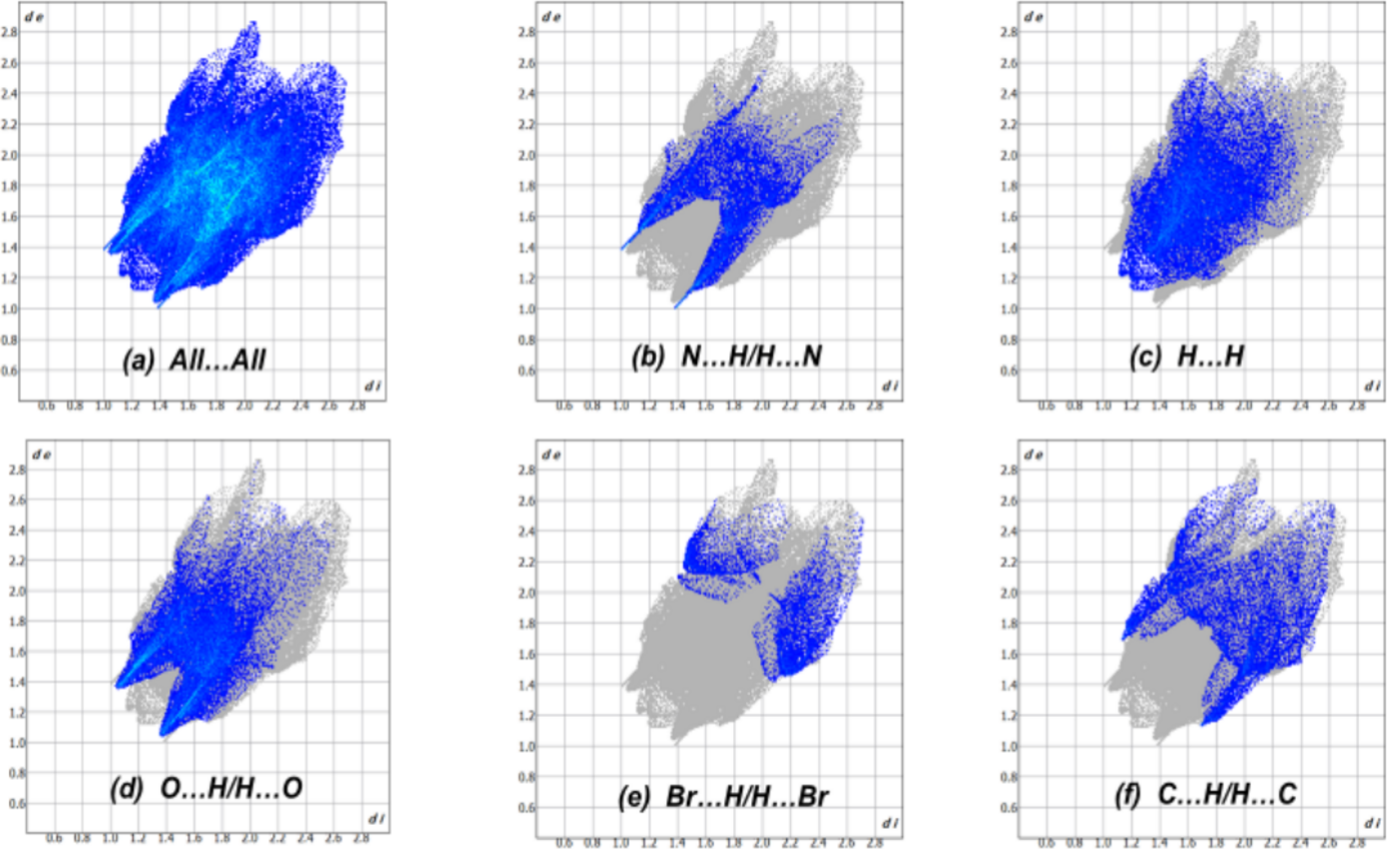
Two-dimensional fingerprint plots of the inter­molecular contacts in **1**.

A Hirshfeld surface analysis was performed using *CrystalExplorer21* (Spackman *et al.*, 2021 ▸). The Hirshfeld surface of mol­ecule **1** mapped over *d*_norm_ is shown in Fig. 3 ▸. The C—H⋯O and C—H⋯N contacts are represented by red spots on the *d*_norm_ surface, indicating close inter­actions (hydrogen bonds). The 2D fingerprint plots (McKinnon *et al.*, 2007 ▸), show that inter­molecular H⋯H and O⋯H/H⋯O contacts make the largest contributions to the total Hirshfeld surface, 23.2% and 25.7%, respectively, other significant contributions being N⋯H/H⋯N (11.7%), Br⋯H/H⋯Br (5.6%) and C⋯H/H⋯C (11.1%) (Fig. 4 ▸). The characteristic ‘spikes’ in the N⋯H/H⋯N and especially O⋯H/H⋯O plots are also indicative of hydrogen bonds.

## 5. Database survey

A search of the Cambridge Structural Database (CSD, Version 5.43, last update November 2022; Groom *et al.*, 2016 ▸) for the 1-(4-bromo­phen­yl)-1*H*-1,2,3-triazole unit, resulted in four hits, CSD refcodes CEWMID (Tireli *et al.*, 2017 ▸), HEHNAL (Boechat *et al.*, 2012 ▸), HOHVAD01 (Li *et al.*, 2015 ▸) and XABPIC (Singh *et al.*, 2013 ▸). In these structures, the dihedral angles between the bromophenyl and triazole rings are comparable to those in the title compound.

## 6. Synthesis and crystallization

**Synthesis of 1.**

1.00 g (5 mmol) of *para*-bromo­phenyl­azide, 1.175 g (5 mmol) of prop-2-yn-1-yl-2-(4-nitro­phen­oxy) acetate, 0.10 g (0.32 mmol) of CuBr and 30 ml of toluene were placed into a flask with a reflux condenser, which was heated on an oil bath at the boiling point of toluene (383 K) for 6 h. The progress of the reaction was monitored by thin-layer chromatography. Over time, a precipitate began to form in the reaction mixture. After 6 h, the reaction was stopped and it was left overnight at room temperature. The precipitate was filtered, dried and recrystallized from ethanol, yielding 1.717 g (79.3%) of **1**, m.p. 417–419 K, *R*_f_ = 0.55 (system benzene:methanol, 10:1). Colourless single crystals suitable for X-ray diffraction analysis were grown from ethanol at room temperature over two weeks.

In the ^1^H NMR spectrum (Fig. S1) of **1** in CDCl_3_ the protons of the methyl­ene groups C8H_2_ and C11H_2_ (see atom numbering in Fig. 1 ▸) showed as 2H singlets at 4.76 and 5.43 ppm, respectively. Protons H1 and H5 of the 4-nitro­phen­oxy group gave a 2H doublet (*J* = 9.35 Hz) at 6.94 ppm, H2 and H4 a 2H doublet at 8.18 ppm (*J* = 9.2 Hz). Protons H22 and H26 of the 4-bromo­phenyl group give a 2H doublet (*J* = 9.1 Hz) at 7.59 ppm, H23 and H25 a 2H doublet at 7.66 ppm (*J* = 9.0 Hz). The sole proton of the triazole moiety shows a singlet signal at 8.03 ppm.

## 7. Refinement

Crystal data, data collection and structure refinement details are summarized in Table 2 ▸. H atoms attached to C were positioned geometrically, with C—H = 0.93 Å for aromatic or C—H = 0.97 Å for methyl­ene C atoms, and were refined as riding with *U*_iso_(H) = 1.2*U*_eq_(C).

## Supplementary Material

Crystal structure: contains datablock(s) I. DOI: 10.1107/S2056989024007436/zv2035sup1.cif

Structure factors: contains datablock(s) I. DOI: 10.1107/S2056989024007436/zv2035Isup3.hkl

Figure S1. 1H NMR spectra of compound 1. DOI: 10.1107/S2056989024007436/zv2035sup4.tif

Supporting information file. DOI: 10.1107/S2056989024007436/zv2035Isup4.cml

CCDC reference: 2322358

Additional supporting information:  crystallographic information; 3D view; checkCIF report

## Figures and Tables

**Table 1:** Hydrogen-bond geometry (Å, °)

*D*—H⋯*A*	*D*—H	H⋯*A*	*D*⋯*A*	*D*—H⋯*A*
C26—H26⋯N17^i^	0.93	2.54	3.463 (3)	173
C5—H5⋯O16^ii^	0.93	2.56	3.493 (3)	176
C8—H8*A*⋯O16^ii^	0.97	2.51	3.195 (3)	128
C8—H8*B*⋯O14^iii^	0.97	2.54	3.427 (3)	153
C2—H2⋯O16^iv^	0.93	2.52	3.287 (3)	140

Symmetry codes: (i) 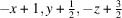; (ii) 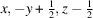; (iii) 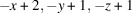; (iv) 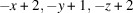.

**Table 2:** Experimental details

Crystal data	
Chemical formula	C_17_H_13_BrN_4_O_5_
*M*_r_	433.22
Crystal system, space group	Monoclinic, *P*2_1_/*c*
Temperature (K)	293
*a*, *b*, *c* (Å)	17.3468 (3), 10.40583 (19), 9.87841 (16)
β (°)	99.4243 (16)
*V* (Å^3^)	1759.07 (5)
*Z*	4
Radiation type	Cu *K*α
μ (mm^−1^)	3.54
Crystal size (mm)	0.6 × 0.4 × 0.2

Data collection	
Diffractometer	XtaLAB Synergy, Single source at home/near, HyPix3000
Absorption correction	Multi-scan (*CrysAlis PRO*; Rigaku OD, 2020 ▸)
*T*_min_, *T*_max_	0.654, 1.000
No. of measured, independent and observed [*I* > 2σ(*I*)] reflections	16655, 3401, 2758
*R*_int_	0.044
(sin θ/λ)_max_ (Å^−1^)	0.616

Refinement	
*R*[*F*^2^ > 2σ(*F*^2^)], *wR*(*F*^2^), *S*	0.035, 0.094, 1.06
No. of reflections	3401
No. of parameters	244
H-atom treatment	H-atom parameters constrained
Δρ_max_, Δρ_min_ (e Å^−3^)	0.27, −0.53

Computer programs: *CrysAlis PRO* (Rigaku OD, 2020 ▸), *SHELXT2014/1* (Sheldrick, 2015*a* ▸), *SHELXL2018/3* (Sheldrick, 2015*b* ▸), *Mercury* (Macrae *et al.*, 2020 ▸) and *publCIF* (Westrip, 2010 ▸).

## supplementary crystallographic information

### [1-(4-Bromophenyl)-1H-1,2,3-triazol-4-yl]methyl 2-(4-nitrophenoxy)acetate Crystal data

**Table:**

C_17_H_13_BrN_4_O_5_	*D*_x_ = 1.636 Mg m^−^^3^
*M**_r_* = 433.22	Melting point: 419 K
Monoclinic, *P*2_1_/*c*	Cu *K*α radiation, λ = 1.54184 Å
*a* = 17.3468 (3) Å	Cell parameters from 5570 reflections
*b* = 10.40583 (19) Å	θ = 2.6–70.8°
*c* = 9.87841 (16) Å	µ = 3.54 mm^−^^1^
β = 99.4243 (16)°	*T* = 293 K
*V* = 1759.07 (5) Å^3^	Prism, colourless
*Z* = 4	0.6 × 0.4 × 0.2 mm
*F*(000) = 872	

### [1-(4-Bromophenyl)-1H-1,2,3-triazol-4-yl]methyl 2-(4-nitrophenoxy)acetate Data collection

**Table:**

XtaLAB Synergy, Single source at home/near, HyPix3000 diffractometer	2758 reflections with *I* > 2σ(*I*)
Detector resolution: 10.0000 pixels mm^-1^	*R*_int_ = 0.044
ω scans	θ_max_ = 71.6°, θ_min_ = 2.6°
Absorption correction: multi-scan (CrysAlisPro; Rigaku OD, 2020)	*h* = −21→21
*T*_min_ = 0.654, *T*_max_ = 1.000	*k* = −12→11
16655 measured reflections	*l* = −11→12
3401 independent reflections	

### [1-(4-Bromophenyl)-1H-1,2,3-triazol-4-yl]methyl 2-(4-nitrophenoxy)acetate Refinement

**Table:**

Refinement on *F*^2^	Primary atom site location: structure-invariant direct methods
Least-squares matrix: full	Secondary atom site location: difference Fourier map
*R*[*F*^2^ > 2σ(*F*^2^)] = 0.035	Hydrogen site location: inferred from neighbouring sites
*wR*(*F*^2^) = 0.094	H-atom parameters constrained
*S* = 1.06	*w* = 1/[σ^2^(*F*_o_^2^) + (0.0412*P*)^2^ + 0.3925*P*] where *P* = (*F*_o_^2^ + 2*F*_c_^2^)/3
3401 reflections	(Δ/σ)_max_ < 0.001
244 parameters	Δρ_max_ = 0.27 e Å^−^^3^
0 restraints	Δρ_min_ = −0.53 e Å^−^^3^

### [1-(4-Bromophenyl)-1H-1,2,3-triazol-4-yl]methyl 2-(4-nitrophenoxy)acetate Special details

**Table:**

Geometry. All esds (except the esd in the dihedral angle between two l.s. planes) are estimated using the full covariance matrix. The cell esds are taken into account individually in the estimation of esds in distances, angles and torsion angles; correlations between esds in cell parameters are only used when they are defined by crystal symmetry. An approximate (isotropic) treatment of cell esds is used for estimating esds involving l.s. planes.

### [1-(4-Bromophenyl)-1H-1,2,3-triazol-4-yl]methyl 2-(4-nitrophenoxy)acetate Fractional atomic coordinates and isotropic or equivalent isotropic displacement parameters (Å^2^)

**Table:**

	*x*	*y*	*z*	*U*_iso_*/*U*_eq_	
Br27	0.23315 (2)	0.34083 (3)	0.13747 (3)	0.05876 (13)	
C25	0.33490 (14)	0.4068 (2)	0.3824 (3)	0.0435 (5)	
H25	0.312633	0.487975	0.368205	0.052*	
O10	0.71928 (9)	0.30539 (17)	0.81015 (16)	0.0444 (4)	
O7	0.92280 (10)	0.35886 (19)	0.85518 (17)	0.0513 (5)	
O16	0.80463 (11)	0.33428 (18)	1.00422 (17)	0.0526 (5)	
N19	0.48779 (11)	0.24275 (18)	0.6241 (2)	0.0393 (4)	
O14	1.15099 (13)	0.4209 (2)	0.4317 (2)	0.0723 (6)	
O15	1.21345 (13)	0.5260 (2)	0.6045 (3)	0.0725 (6)	
N18	0.50303 (15)	0.1230 (2)	0.6747 (3)	0.0599 (6)	
N13	1.15860 (13)	0.4594 (2)	0.5506 (3)	0.0531 (6)	
N17	0.56280 (15)	0.1328 (2)	0.7739 (3)	0.0629 (7)	
C24	0.31006 (14)	0.3080 (2)	0.2928 (2)	0.0423 (5)	
C21	0.42602 (13)	0.2639 (2)	0.5120 (2)	0.0377 (5)	
C26	0.39253 (14)	0.3849 (2)	0.4926 (3)	0.0413 (5)	
H26	0.409094	0.450859	0.554001	0.050*	
C9	0.79074 (14)	0.3230 (2)	0.8828 (2)	0.0382 (5)	
C3	1.09852 (14)	0.4243 (2)	0.6322 (3)	0.0449 (6)	
C6	0.97995 (14)	0.3746 (2)	0.7767 (2)	0.0424 (5)	
C20	0.53775 (14)	0.3271 (2)	0.6942 (3)	0.0429 (5)	
H20	0.538958	0.415583	0.681321	0.052*	
C12	0.58587 (14)	0.2570 (2)	0.7874 (3)	0.0434 (6)	
C5	0.97409 (15)	0.3327 (3)	0.6420 (3)	0.0463 (6)	
H5	0.930095	0.287661	0.600952	0.056*	
C8	0.84817 (14)	0.3220 (3)	0.7848 (2)	0.0441 (6)	
H8A	0.850981	0.236529	0.746602	0.053*	
H8B	0.831325	0.381126	0.709912	0.053*	
C22	0.40085 (15)	0.1648 (2)	0.4226 (3)	0.0441 (6)	
H22	0.423542	0.083905	0.436396	0.053*	
C23	0.34185 (16)	0.1861 (2)	0.3127 (3)	0.0466 (6)	
H23	0.323799	0.119527	0.252980	0.056*	
C11	0.65520 (15)	0.2966 (3)	0.8875 (3)	0.0505 (6)	
H11A	0.646112	0.379063	0.927913	0.061*	
H11B	0.666662	0.233527	0.960297	0.061*	
C2	1.10697 (15)	0.4614 (3)	0.7680 (3)	0.0503 (6)	
H2	1.152186	0.502689	0.809906	0.060*	
C4	1.03377 (15)	0.3580 (2)	0.5696 (3)	0.0470 (6)	
H4	1.030331	0.330596	0.479185	0.056*	
C1	1.04753 (15)	0.4362 (3)	0.8399 (3)	0.0521 (7)	
H1	1.052372	0.460485	0.931496	0.063*	

### [1-(4-Bromophenyl)-1H-1,2,3-triazol-4-yl]methyl 2-(4-nitrophenoxy)acetate Atomic displacement parameters (Å^2^)

**Table:**

	*U*^11^	*U*^22^	*U*^33^	*U*^12^	*U*^13^	*U*^23^
Br27	0.0594 (2)	0.0613 (2)	0.05047 (19)	−0.00121 (14)	−0.00636 (14)	−0.00058 (13)
C25	0.0436 (13)	0.0356 (12)	0.0504 (14)	0.0032 (10)	0.0049 (11)	0.0015 (11)
O10	0.0324 (8)	0.0594 (11)	0.0399 (9)	−0.0027 (7)	0.0020 (7)	0.0041 (8)
O7	0.0361 (9)	0.0798 (13)	0.0372 (9)	−0.0098 (8)	0.0034 (7)	−0.0045 (8)
O16	0.0471 (10)	0.0738 (13)	0.0352 (9)	−0.0019 (9)	0.0019 (8)	0.0010 (8)
N19	0.0348 (10)	0.0323 (10)	0.0506 (11)	0.0024 (8)	0.0067 (9)	0.0047 (9)
O14	0.0652 (14)	0.1000 (18)	0.0563 (13)	0.0045 (12)	0.0239 (11)	0.0074 (12)
O15	0.0580 (13)	0.0667 (14)	0.0988 (16)	−0.0162 (11)	0.0307 (12)	−0.0042 (12)
N18	0.0595 (15)	0.0357 (11)	0.0773 (17)	−0.0009 (10)	−0.0099 (12)	0.0114 (11)
N13	0.0463 (13)	0.0515 (13)	0.0643 (15)	0.0071 (10)	0.0171 (11)	0.0111 (11)
N17	0.0587 (15)	0.0465 (13)	0.0766 (17)	0.0007 (11)	−0.0093 (13)	0.0150 (12)
C24	0.0385 (13)	0.0472 (14)	0.0414 (13)	−0.0027 (10)	0.0070 (10)	0.0002 (11)
C21	0.0331 (12)	0.0379 (12)	0.0429 (13)	−0.0016 (9)	0.0090 (10)	0.0016 (10)
C26	0.0402 (13)	0.0347 (12)	0.0484 (14)	−0.0008 (10)	0.0057 (10)	−0.0034 (10)
C9	0.0350 (12)	0.0378 (12)	0.0403 (13)	0.0009 (9)	0.0014 (10)	0.0057 (10)
C3	0.0389 (13)	0.0449 (14)	0.0519 (14)	0.0052 (11)	0.0104 (11)	0.0037 (11)
C6	0.0349 (12)	0.0521 (14)	0.0400 (13)	0.0002 (10)	0.0059 (10)	0.0004 (11)
C20	0.0354 (12)	0.0362 (12)	0.0567 (15)	−0.0028 (10)	0.0058 (11)	0.0029 (11)
C12	0.0333 (12)	0.0481 (14)	0.0496 (14)	0.0001 (10)	0.0094 (10)	0.0033 (11)
C5	0.0384 (13)	0.0558 (16)	0.0430 (13)	−0.0045 (11)	0.0020 (10)	−0.0057 (11)
C8	0.0365 (13)	0.0562 (15)	0.0377 (12)	−0.0058 (11)	0.0007 (10)	−0.0024 (11)
C22	0.0446 (13)	0.0359 (13)	0.0529 (14)	0.0034 (10)	0.0115 (11)	−0.0036 (11)
C23	0.0519 (15)	0.0425 (14)	0.0455 (14)	−0.0045 (11)	0.0084 (12)	−0.0093 (11)
C11	0.0380 (13)	0.0693 (17)	0.0448 (14)	0.0007 (12)	0.0086 (11)	0.0038 (13)
C2	0.0368 (13)	0.0553 (16)	0.0576 (16)	−0.0071 (11)	0.0040 (11)	−0.0067 (13)
C4	0.0441 (14)	0.0547 (16)	0.0422 (13)	0.0015 (12)	0.0071 (11)	−0.0054 (11)
C1	0.0438 (14)	0.0692 (18)	0.0430 (14)	−0.0045 (13)	0.0061 (11)	−0.0098 (13)

### [1-(4-Bromophenyl)-1H-1,2,3-triazol-4-yl]methyl 2-(4-nitrophenoxy)acetate Geometric parameters (Å, º)

**Table:**

Br27—C24	1.892 (2)	C9—C8	1.498 (3)
C25—H25	0.9300	C3—C2	1.381 (4)
C25—C24	1.379 (3)	C3—C4	1.376 (4)
C25—C26	1.371 (3)	C6—C5	1.388 (3)
O10—C9	1.339 (3)	C6—C1	1.391 (3)
O10—C11	1.451 (3)	C20—H20	0.9300
O7—C6	1.365 (3)	C20—C12	1.351 (3)
O7—C8	1.418 (3)	C12—C11	1.485 (4)
O16—C9	1.190 (3)	C5—H5	0.9300
N19—N18	1.353 (3)	C5—C4	1.377 (4)
N19—C21	1.426 (3)	C8—H8A	0.9700
N19—C20	1.344 (3)	C8—H8B	0.9700
O14—N13	1.228 (3)	C22—H22	0.9300
O15—N13	1.226 (3)	C22—C23	1.383 (4)
N18—N17	1.309 (3)	C23—H23	0.9300
N13—C3	1.464 (3)	C11—H11A	0.9700
N17—C12	1.353 (3)	C11—H11B	0.9700
C24—C23	1.385 (4)	C2—H2	0.9300
C21—C26	1.387 (3)	C2—C1	1.370 (3)
C21—C22	1.381 (3)	C4—H4	0.9300
C26—H26	0.9300	C1—H1	0.9300
			
C24—C25—H25	120.1	N17—C12—C11	121.7 (2)
C26—C25—H25	120.1	C20—C12—N17	108.0 (2)
C26—C25—C24	119.8 (2)	C20—C12—C11	130.3 (2)
C9—O10—C11	116.63 (19)	C6—C5—H5	120.2
C6—O7—C8	116.33 (18)	C4—C5—C6	119.6 (2)
N18—N19—C21	120.4 (2)	C4—C5—H5	120.2
C20—N19—N18	109.9 (2)	O7—C8—C9	109.35 (19)
C20—N19—C21	129.7 (2)	O7—C8—H8A	109.8
N17—N18—N19	106.7 (2)	O7—C8—H8B	109.8
O14—N13—C3	118.1 (2)	C9—C8—H8A	109.8
O15—N13—O14	123.6 (2)	C9—C8—H8B	109.8
O15—N13—C3	118.3 (2)	H8A—C8—H8B	108.3
N18—N17—C12	109.5 (2)	C21—C22—H22	120.0
C25—C24—Br27	119.41 (19)	C21—C22—C23	119.9 (2)
C25—C24—C23	121.1 (2)	C23—C22—H22	120.0
C23—C24—Br27	119.47 (19)	C24—C23—H23	120.5
C26—C21—N19	119.5 (2)	C22—C23—C24	119.0 (2)
C22—C21—N19	120.0 (2)	C22—C23—H23	120.5
C22—C21—C26	120.5 (2)	O10—C11—C12	105.9 (2)
C25—C26—C21	119.7 (2)	O10—C11—H11A	110.6
C25—C26—H26	120.2	O10—C11—H11B	110.6
C21—C26—H26	120.2	C12—C11—H11A	110.6
O10—C9—C8	107.9 (2)	C12—C11—H11B	110.6
O16—C9—O10	124.8 (2)	H11A—C11—H11B	108.7
O16—C9—C8	127.3 (2)	C3—C2—H2	120.6
C2—C3—N13	119.6 (2)	C1—C2—C3	118.8 (2)
C4—C3—N13	118.7 (2)	C1—C2—H2	120.6
C4—C3—C2	121.7 (2)	C3—C4—C5	119.4 (2)
O7—C6—C5	124.1 (2)	C3—C4—H4	120.3
O7—C6—C1	115.9 (2)	C5—C4—H4	120.3
C5—C6—C1	120.0 (2)	C6—C1—H1	119.8
N19—C20—H20	127.1	C2—C1—C6	120.4 (2)
N19—C20—C12	105.8 (2)	C2—C1—H1	119.8
C12—C20—H20	127.1		
			
Br27—C24—C23—C22	176.72 (19)	C21—N19—N18—N17	178.9 (2)
C25—C24—C23—C22	−1.7 (4)	C21—N19—C20—C12	−178.4 (2)
O10—C9—C8—O7	171.6 (2)	C21—C22—C23—C24	1.3 (4)
O7—C6—C5—C4	177.3 (2)	C26—C25—C24—Br27	−177.75 (18)
O7—C6—C1—C2	−177.3 (2)	C26—C25—C24—C23	0.6 (4)
O16—C9—C8—O7	−10.0 (4)	C26—C21—C22—C23	0.1 (4)
N19—N18—N17—C12	0.1 (3)	C9—O10—C11—C12	−171.2 (2)
N19—C21—C26—C25	177.9 (2)	C3—C2—C1—C6	−0.1 (4)
N19—C21—C22—C23	−178.9 (2)	C6—O7—C8—C9	−173.9 (2)
N19—C20—C12—N17	−1.5 (3)	C6—C5—C4—C3	0.2 (4)
N19—C20—C12—C11	176.0 (2)	C20—N19—N18—N17	−1.0 (3)
O14—N13—C3—C2	177.3 (3)	C20—N19—C21—C26	−23.2 (4)
O14—N13—C3—C4	−4.5 (4)	C20—N19—C21—C22	155.8 (2)
O15—N13—C3—C2	−3.1 (4)	C20—C12—C11—O10	−79.9 (3)
O15—N13—C3—C4	175.0 (2)	C5—C6—C1—C2	3.2 (4)
N18—N19—C21—C26	156.9 (2)	C8—O7—C6—C5	−13.6 (4)
N18—N19—C21—C22	−24.1 (3)	C8—O7—C6—C1	166.9 (2)
N18—N19—C20—C12	1.6 (3)	C22—C21—C26—C25	−1.1 (3)
N18—N17—C12—C20	0.9 (3)	C11—O10—C9—O16	−1.6 (4)
N18—N17—C12—C11	−176.8 (2)	C11—O10—C9—C8	176.9 (2)
N13—C3—C2—C1	175.2 (2)	C2—C3—C4—C5	2.9 (4)
N13—C3—C4—C5	−175.2 (2)	C4—C3—C2—C1	−3.0 (4)
N17—C12—C11—O10	97.3 (3)	C1—C6—C5—C4	−3.2 (4)
C24—C25—C26—C21	0.8 (4)		

### [1-(4-Bromophenyl)-1H-1,2,3-triazol-4-yl]methyl 2-(4-nitrophenoxy)acetate Hydrogen-bond geometry (Å, º)

**Table:**

*D*—H···*A*	*D*—H	H···*A*	*D*···*A*	*D*—H···*A*
C26—H26···N17^i^	0.93	2.54	3.463 (3)	173
C5—H5···O16^ii^	0.93	2.56	3.493 (3)	176
C8—H8*A*···O16^ii^	0.97	2.51	3.195 (3)	128
C8—H8*B*···O14^iii^	0.97	2.54	3.427 (3)	153
C2—H2···O16^iv^	0.93	2.52	3.287 (3)	140

Symmetry codes: (i) −*x*+1, *y*+1/2, −*z*+3/2; (ii) *x*, −*y*+1/2, *z*−1/2; (iii) −*x*+2, −*y*+1, −*z*+1; (iv) −*x*+2, −*y*+1, −*z*+2.

## References

[bb1] Bertrand, G. & Wentrup, C. (1994). *Angew. Chem. Int. Ed. Engl.* **33**, 527–545.

[bb2] Boechat, N., de Lourdes, G., Ferreira, M., Bastos, M. M., da Silva, G. P., Wardell, J. & Wardell, J. (2012). *Z. Kristallogr.* **227**, 369–378.

[bb3] Borgati, Th. F., Alves, R. B., Teixeira, R. R., Freitas, R. P., Perdigão, Th. G., Silva, S. F., Santos, A. A. & Bastidas, A. J. O. (2013). *J. Braz. Chem. Soc.* **24**, 953–961.

[bb4] Bozorov, Kh., Zhao, J. & Aisa, H. A. (2019). *Bioorg. Med. Chem.* **27**, 3511–3531. 10.1016/j.bmc.2019.07.005 PMC7185471 31300317

[bb5] Faraz, Kh. M., Garima, V., Wasim, A., Akranth, M., Mumtoz, A. M., Mymoona, A., Asif, H., Misbahul, H. S., Mohammad, Sh. & Rashiduddin, H. S. (2017). *Int. J. Drug Dev. Res*, **9**(2), 22–25.

[bb6] Groom, C. R., Bruno, I. J., Lightfoot, M. P. & Ward, S. C. (2016). *Acta Cryst.* B**72**, 171–179. 10.1107/S2052520616003954 PMC4822653 27048719

[bb7] Huisgen, R. (1963). *Angew. Chem. Int. Ed. Engl.* **2**, 565–598.

[bb8] Li, W., Zhou, X., Luan, Y. & Wang, J. (2015). *RSC Adv.* **5**, 88816–88820.

[bb9] Macrae, C. F., Sovago, I., Cottrell, S. J., Galek, P. T. A., McCabe, P., Pidcock, E., Platings, M., Shields, G. P., Stevens, J. S., Towler, M. & Wood, P. A. (2020). *J. Appl. Cryst.* **53**, 226–235. 10.1107/S1600576719014092 PMC6998782 32047413

[bb10] McKinnon, J. J., Jayatilaka, D. & Spackman, M. A. (2007). *Chem. Commun.* pp. 3814–3816. 10.1039/b704980c 18217656

[bb11] Rigaku OD (2020). *CrysAlis PRO*. Rigaku Oxford Diffraction, Yarnton, England.

[bb12] Rostovtsev, V. V., Green, L. G., Fokin, V. V. & Sharpless, K. B. (2002). *Angew. Chem. Int. Ed.* **41**, 2596–2599. 10.1002/1521-3773(20020715)41:14<2596::AID-ANIE2596>3.0.CO;2-4 12203546

[bb13] Sheldrick, G. M. (2015*a*). *Acta Cryst.* A**71**, 3–8.

[bb14] Sheldrick, G. M. (2015*b*). *Acta Cryst.* C**71**, 3–8.

[bb15] Singh, H., Sindhu, J. & Khurana, J. M. (2013). *J. Iran. Chem. Soc.* **10**, 883–888.

[bb16] Spackman, P. R., Turner, M. J., McKinnon, J. J., Wolff, S. K., Grimwood, D. J., Jayatilaka, D. & Spackman, M. A. (2021). *J. Appl. Cryst.* **54**, 1006–1011. 10.1107/S1600576721002910 PMC8202033 34188619

[bb17] Tireli, M., Maračić, S., Lukin, S., Kulcsár, M. J., Žilić, D., Cetina, M., Halasz, I., Raić-Malić, S. & Užarević, K. (2017). *Beilstein J. Org. Chem.* **13**, 2352–2363. 10.3762/bjoc.13.232 PMC5687011 29181115

[bb18] Tornøe, Ch. W., Christensen, C. & Meldal, M. (2002). *J. Org. Chem.* **67**, 3057–3064. 10.1021/jo011148j 11975567

[bb19] Westrip, S. P. (2010). *J. Appl. Cryst.* **43**, 920–925.

