# Supplementary figures and images for: Synthesis, crystal structure and Hirshfeld surface analysis of [1-(4-bromo­phen­yl)-1H-1,2,3-triazol-4-yl]methyl 2-(4-nitro­phen­oxy)acetate
Source: Acta Crystallogr E Crystallogr Commun. 2024 Jul 31;80(Pt 8):910–2. doi: 10.1107/S2056989024007436 (PMC11299749; doi:10.1107/S2056989024007436)

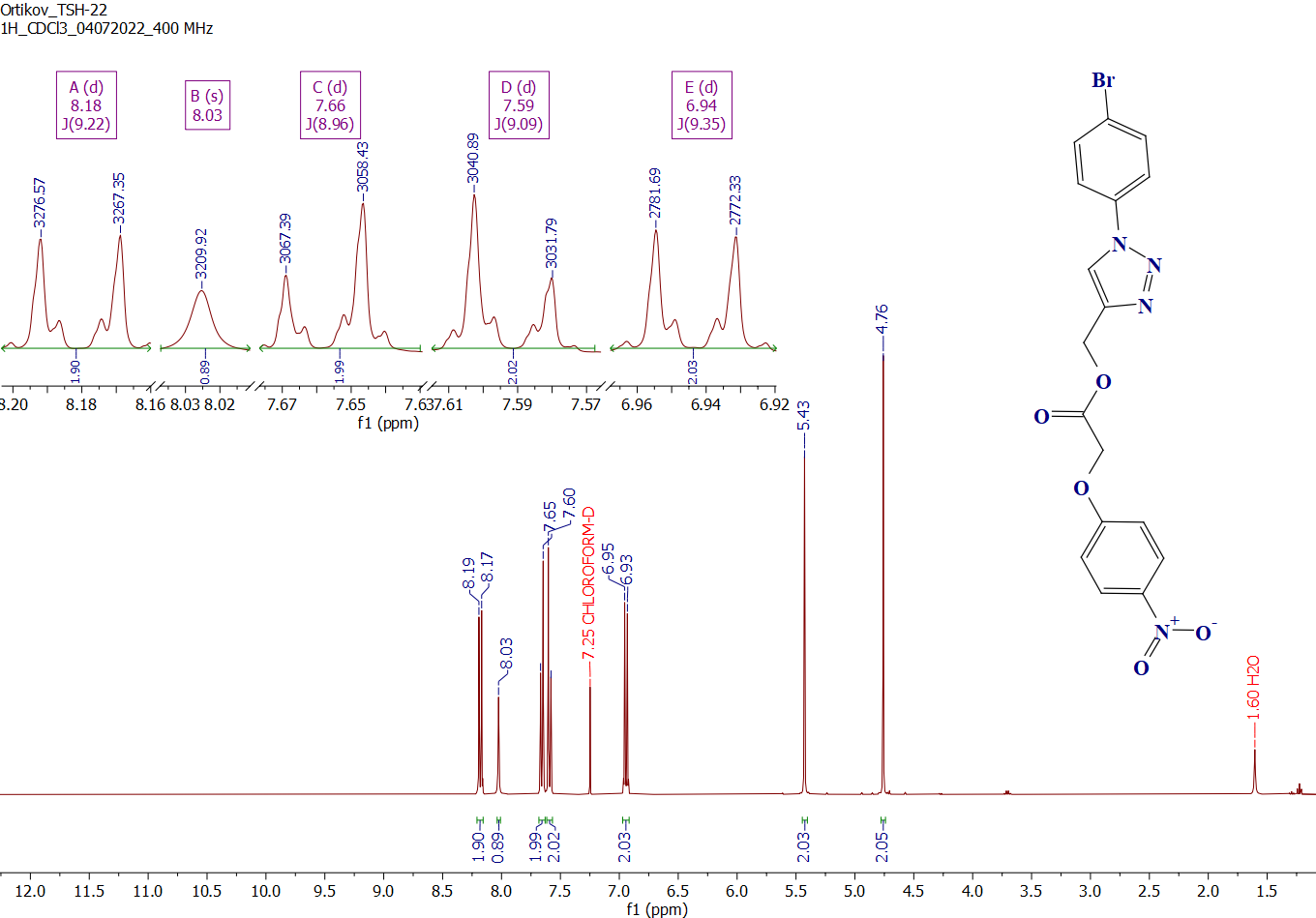

Supplement: Supplementary file 3 [file e-80-00910-sup4.tif]
